# The Class I HD-ZIP transcription factor PagHB7a functions as a positive regulator of salt tolerance in *Populus*

**DOI:** 10.48130/forres-0025-0030

**Published:** 2025-12-31

**Authors:** Bowen Zhou, Na Xu, Zhuoran Yang, Xingkai Sun, Yihao Sun, Zhenyang Ji, Li-Jun Liu

**Affiliations:** State Forestry and Grassland Administration Key Laboratory of Silviculture in downstream areas of the Yellow River, College of Forestry, Shandong Agricultural University, Taian, Shandong 271018, China

**Keywords:** Class I HD-ZIP, PagHB7a, Salt tolerance, *Populus*

## Abstract

Homeodomain leucine zipper (HD-ZIP) proteins are plant-specific transcription factors that play important roles in plant development and abiotic responses. In our previous study, the *PagHB7a* gene was identified, which belongs to the Class I HD ZIP family, and was among the most significantly induced genes by salt stress in poplar. In the present study, the role of *PagHB7a* was functionally characterized in salt stress responses. Expression analysis confirmed that *PagHB7a* was significantly induced by salt and abscisic acid (ABA) treatments; moreover, *PagHB7a* was directly regulated by the ABA-responsive element (ABRE) binding proteins (PagAREB1s). Genetic analysis showed that overexpression of *PagHB7a* (*PagHB7a-OE*) significantly enhanced salt tolerance, whereas CRISPR/Cas9-mediated knockout of *PagHB7a* (*PagHB7a-KO*) significantly reduced it. Transcriptome analysis revealed that biological pathways responding to salt stress, ABA, and oxidative stress were significantly upregulated in *PagHB7a-OE* plants. Collectively, our results demonstrate that PagHB7a, a salt stress- and ABA-inducible transcription factor, acts as a positive regulator of salt tolerance in *Populus*.

## Introduction

Soil salinization is a major environmental constraint that severely limits plant growth, and reduces agricultural and forestry productivity worldwide^[1−3]^. It is estimated that approximately 800 million hectares of global land area, accounting for about 6% of total terrestrial land, are affected by excessive salt concentrations, with salinity impacting nearly 20% of irrigated arable land. These saline-affected areas continue to expand due to climate change and unsustainable irrigation practices^[2,4]^. Under salt stress, plants experience secondary stresses including osmotic imbalance, ion toxicity, and oxidative damage, which collectively disrupt metabolic homeostasis, photosynthetic efficiency, and developmental processes^[5]^. Therefore, elucidating the genetic mechanisms and signaling networks underlying salt tolerance in woody species is critical for developing stress-resilient cultivars and mitigating productivity losses in marginal environments.

Salt stress induces ionic toxicity in plant cells by disrupting the Na^+^/K^+^ ratio, which impairs physiological functions, compromises metabolic homeostasis, and inhibits growth^[6,7]^. Concurrently, high salinity triggers the excessive accumulation of reactive oxygen species (ROS) such as hydroxyl radicals (·OH), hydrogen peroxide (H_2_O_2_), and superoxide anions (O_2_·^−^) within plant cells^[8−11]^. This oxidative burst promotes lipid peroxidation, damaging cellular components such as nucleic acids, proteins, and lipids, and can ultimately lead to cell death^[12−14]^. To cope with prolonged stress, plants have evolved sophisticated adaptive mechanisms involving coordinated physiological, biochemical, and signaling pathways^[2,15]^. Among these mechanisms, enzymatic antioxidants play a critical role in scavenging excess ROS and maintaining normal growth under stressful conditions^[16−18]^.

Abscisic acid (ABA) is a key phytohormone that mediates stress signaling and promotes sodium ion efflux and water uptake under salt stress conditions^[19−23]^. AREB/ABF transcription factors are members of the bZIP family that function in the ABA signaling pathway. They bind to ABA-responsive elements (ABRE: PyACGTGG/TC) in the promoters of stress-responsive genes, thereby activating their expression to enhance abiotic stress tolerance. The role of AREB transcription factors in regulating drought and salt stress responses is well established. For instance, transgenic Arabidopsis plants overexpressing *AREB1*/*ABF2*, *AREB2*/*ABF4*, or *ABF3* exhibit enhanced drought tolerance^[23]^. In poplar, PtrAREB1-2 binds to the ABRE motifs in drought-responsive genes such as *PtrNAC* and *PtrHox*, playing a critical role in their transcriptional activation under drought conditions in *Populus*^[24−26]^. Furthermore, PagAREB1-3 has been identified as a positive regulator of salt tolerance, and the PagAFP2a-PagAREB1s module forms a negative feedback loop in ABA signaling that fine-tunes salt stress responses in *Populus*^[27]^*.*

At the transcriptional level, stress adaptation is coordinated by an intricate regulatory network involving multiple transcription factor families, including HD-ZIP, bZIP, MYB, NAC, WRKY, and AP2/ERF^[26,28−33]^, which collectively modulate stress-responsive gene expression and signal transduction pathways. Among these, the homeodomain-leucine zipper (HD-ZIP) family represents a plant-specific group of transcription factors characterized by a homeodomain (HD) responsible for DNA binding and an adjacent leucine zipper (LZ) motif that facilitates protein dimerization^[34]^. Phylogenetic analyses classify HD-ZIP proteins into four distinct subfamilies (HD-ZIP I-IV) based on sequence homology, gene structure, and functional specialization^[35,36]^. As one of the largest subfamilies, HD-ZIP I members orchestrate crucial regulatory functions in abiotic stress responses, hormonal signaling cascades, and developmental processes^[37−39]^. Notably, several HD-ZIP I members have been closely linked to stress adaptation. In Arabidopsis, *ATHB7* and *ATHB12* are upregulated by ABA, water deficit, and osmotic stress^[40,41]^, while ATHB6 interacts with the protein phosphatase ABI1 to modulate ABA signaling^[42]^. In *Medicago truncatula*, *MtHB1* is induced by salt stress and expressed in root meristems, suggesting its potential role in stress adaptation and root development^[36]^. Overexpression of *MdHB7*, as in apple, enhances salt tolerance by improving photosynthetic performance, reducing ROS and Na^+^ accumulation, and promoting osmolyte synthesis^[43]^. Similarly, in poplar, PagHB7 interacts with PagABF4 to negatively regulate *PagEPFL9* expression, thereby reducing stomatal density and enhancing drought tolerance^[28]^. These findings underscore the pivotal and evolutionarily conserved role of HD-ZIP I transcription factors, particularly HB7 homologs, in coordinating adaptive responses to abiotic stresses.

Populus is both an ecologically important genus and a valuable economic resource, serving as a model system for studying stress adaptation mechanisms in woody plants. However, soil salinity has become a critical factor that severely limits poplar growth and productivity^[44]^. In this study, we characterized the function of PagHB7a in poplar was characterized, and its essential role demonstrated in salt stress response. *PagHB7a* expression was significantly induced by salt stress and ABA treatment, and PagAREB1s were identified as upstream transcriptional activators of *PagHB7a* under salt stress. Genetic analysis with *PagHB7* overexpression or knockout plants demonstrated that it is a positive regulator of salt tolerance. Transcriptome profiling further corroborated these findings and identified the differentially expressed genes in *PagHB7* overexpression plants. Collectively, our results demonstrate that PagHB7a enhances salt tolerance in poplar by maintaining redox homeostasis, providing new insights into the molecular mechanisms of stress adaptation in woody plants.

## Materials and methods

### Plant material and growth conditions

The hybrid poplar (*Populus alba* × *Populus glandulosa*) clone 84K was used in this study. Poplar cuttings were propagated through tissue culture on Murashige and Skoog (MS) medium. All plants were cultivated in a controlled growth chamber under a 16 h light/8 h dark cycle at 25 °C.

To examine the tissue-specific expression of *PagHB7a* in poplar, stem, leaf, root, and petiole samples were collected from 2-month-old wild-type (WT) plants grown in a growth chamber. The expression pattern of *PagHB7a* in response to NaCl and ABA was analyzed in 45-day-old 84K poplar seedlings using previously described methods with minor modifications^[45,46]^. For NaCl treatment, 20-day-old WT plants cultivated in 1/4 Hoagland’s solution were transferred to fresh 1/4 Hoagland’s solution supplemented with 100 mM NaCl. Root tissues were harvested after 0, 1, 3, 6, 12, 24, and 48 h of treatment for RT-qPCR analysis. For ABA treatment, 20-day-old poplar plants maintained in 1/4 Hoagland’s solution were sprayed with 200 μM ABA solution containing 0.1% (v/v) TWEEN 20^[45]^. Leaf samples were collected at 0, 1, 3, 6, 12, and 24 h post-treatment for subsequent RT-qPCR. The primers used are listed in Supplementary Table S1.

Salt stress treatments were applied as previously described^[27]^. For short-term NaCl treatment, 1-month-old plants were pre-cultured in 1/4 Hoagland’s solution for 20 d, and then exposed to 150 mM NaCl solution for 2 d. At the end of the treatment, two leaves from the same nodal position on three individual plants were collected, immediately frozen in liquid nitrogen, and used for subsequent physiological assays. For long-term salt stress evaluation, 1-month-old plants were transplanted into soil and grown for 30 d, followed by irrigation with 100 mM NaCl solution for 20 d. Growth and biomass parameters were measured at the end of the treatment period, with 6–12 plants analyzed per genotype in each experiment.

To assess ABA sensitivity, seven-day-old poplar seedlings were transplanted onto MS medium supplemented with 5 μM ABA for 23 d^[46]^. After the treatment, growth phenotypes were documented, root fresh weight was measured, and root length was quantified using ImageJ software. Leaf tissues were harvested after ABA treatment for RT-qPCR analysis. Four plants of each genotype were used in the experiment. The primers used are listed in Supplementary Table S1.

### Gene cloning and plant transformation

To construct the *Pro35S::PagHB7a* vector, the full-length coding sequence (CDS) of *PagHB7a* was amplified from 84K poplar cDNA using gene-specific primers, and cloned into the PzP211-35S-PolyA binary vector for expression under the control of the *CaMV 35S* promoter. For the *ProPagHB7a::GUS* reporter construct, a 2,000 bp fragment upstream of the *PagHB7a* start codon was amplified from 84K poplar genomic DNA, and inserted into the pBI121 vector to drive *β*-glucuronidase (*GUS*) expression. The *Pro35S::PagAREB1-3‐GFP* overexpression construct was generated by fusing the full-length CDS of *PagAREB1-3* (without the stop codon) into the pROKII-GFP vector, also under the control of the *CaMV 35S* promoter. For CRISPR/Cas9-mediated mutagenesis, two sgRNAs targeting *PagHB7a* were designed using the web tool (http://crispr.dbcls.jp) and assembled into the binary pYLCRISPR/Cas9 vector, according to established protocols^[47]^. All constructs were introduced into 84K poplar via Agrobacterium-mediated transformation as described previously^[48]^. The primers used are listed in Supplementary Table S1.

### RNA extraction and real-time quantitative PCR (RT-qPCR)

Total RNA was extracted from 84K poplar plants using the FastPure Universal Plant Total RNA Isolation Kit (Vazyme, RC411-01). Subsequently, 1 μg of total RNA was reverse-transcribed into cDNA using the HiScript II Q RT SuperMix for qPCR Kit (Vazyme, R233-01). Quantitative real-time PCR (RT-qPCR) was performed with ChamQ SYBR Color qPCR Master Mix (Vazyme, Q312-02), using PagActin (Potri.006G192700) as an internal reference gene. Gene expression levels were normalized and calculated using the 2^−ΔΔCq^ method^[49]^. At least three biological replicates were performed. The primers used for RT-qPCR are listed in Supplementary Table S1.

### Subcellular localization analysis

To examine the subcellular localization of PagHB7a, the full-length CDS of *PagHB7a* (without the stop codon) was cloned into the pROKII-GFP vector to generate a C-terminal GFP fusion. The resulting *Pro35S::PagHB7a-GFP* construct, and an empty pROKII-GFP control vector, were separately transfected into 84K poplar protoplasts. Nuclei were counterstained with DAPI. Transfected protoplasts were observed under an LSM880 confocal microscope, with GFP fluorescence detected at 488 nm. Transient transfection of protoplasts was performed according to a previously established method^[50]^. The primers used are listed in Supplementary Table S1.

### GUS staining

The positive GUS reporter lines driven by the promoter of *PagHB7a* (*ProPagHB7a::GUS*) were cultivated on MS solid medium for 4 weeks. To evaluate salt-induced expression, primary roots of 4-week-old *ProPagHB7a::GUS* plants were treated with 100 mM NaCl solution, and harvested at 0 and 6 h for GUS staining, as previously described^[51]^. Histochemical staining was performed using a *β*-Glucuronidase Reporter Gene Staining Kit (Coolaber, SL7160, China), according to the manufacturer's protocol. To analyze the expression of the GUS gene driven by the *PagHB7a* promoter under salt stress, 4-week-old *ProPagHB7a::GUS* transgenic plants were grown in 1/4 Hoagland’s solution for 10 d before being transferred to 100 mM NaCl solution. Roots were harvested at 0 and 6 h after treatment for RT-qPCR analysis. Three independent *ProPagHB7a::GUS* plants were used for staining and RT-qPCR analysis.

### Physiological analysis

Salt-treated poplar leaf tissues were harvested and ground in liquid nitrogen. Catalase (CAT) and peroxidase (POD) activities, as well as malondialdehyde (MDA) content, were measured using commercial assay kits (Suzhou Keming Biotechnology, China) following the manufacturer's instructions. Hydrogen peroxide (H_2_O_2_), and superoxide anion (O_2_·^−^) levels were quantified with corresponding detection kits (Solarbio, China). For histochemical staining, fresh leaf discs (0.6 mm in diameter) were harvested and stained following the kit manufacturer's protocols (Solarbio, China). Staining with 3,3′-diaminobenzidine (DAB) was performed for 12 h, and with nitroblue tetrazolium (NBT) for 6 h, both at 30 °C in the dark. The presented images are representative of results obtained from at least three independent biological replicates. Relative conductivity (REC), relative water content (RWC), fresh weight (FW), dry weight (DW), and chlorophyll content were determined using previously described methods^[27]^.

### Yeast one-hybrid (Y1H) assay

The ABRE cis-element-containing sequences derived from the *PagHB7a* promoter were cloned and inserted into the pLacZi2u vector^[52]^. The full-length CDS of *PagAREB1-2*, *PagAREB1-3*, *PagAREB1-4*, and *PagABF3* were cloned into the pJG4-5 vector to generate *AD-PagAREB1-2*/*PagAREB1-3*/*PagAREB1-4*/*PagABF3* construct, respectively. Various combinations of these recombinant constructs were co-transformed into yeast EGY48 cells. Transformants were selected on synthetic dropout (SD) medium lacking tryptophan and uracil (SD/–Trp/–Ura) and incubated at 30  °C for 2–3 d. Positive colonies were cultured overnight in liquid medium and then transferred to SD/–Trp/–Ura solid medium containing raffinose, galactose, and X-gal (40 mg/L) for chromogenic detection of interactions over 48 to 72 h. Each experiment included at least three biological replicates. The primers used are listed in Supplementary Table S1.

### Dual-luciferase assay

The full-length coding sequences of *PagAREB1-2*, *PagAREB1-3*, *PagAREB1-4*, and *PagABF3* were cloned into the PzP211-35S-PolyA vector to generate effector constructs^[53]^. The promoter region of *PagHB7* containing ABRE *cis*-elements was inserted into the pGreenII0800−LUC vector to create the reporter construct^[53]^. Each construct was introduced into *Agrobacterium tumefaciens* strain GV3101 (pSoup-p19). Bacterial cultures were resuspended in infiltration buffer (10 mM MES, pH 5.6, 1 mM MgCl_2_, 100 μM acetosyringone), adjusted to an OD_600_ of 1.0, and mixed at a 1:1 ratio (effector:reporter) before co-infiltration into leaves of *Nicotiana benthamiana*^[54]^. After 72 h of transient expression, firefly and *Renilla* luciferase activities were measured using a dual-luciferase assay kit (Vazyme, DL101-01, China). Three independent biological replicates were performed for each experiment. The primers used are listed in Supplementary Table S1.

### Chromatin immunoprecipitation (ChIP)-qPCR

Leaf and stem samples from 1-month-old *PagAREB1-3-GFP* overexpression poplar plants were collected and cross-linked in 1% formaldehyde under vacuum before being ground into a fine powder in liquid nitrogen. Nuclei were isolated using the CelLytic™ PN extraction kit (Sigma-Aldrich). Chromatin was fragmented into 300–500 bp pieces via sonication in lysis buffer (50 mM Tris-HCl pH 8.0, 10 mM EDTA, 0.3% SDS, 0.5% sodium deoxycholate, supplemented with protease inhibitors and 1 mM PMSF). Chromatin immunoprecipitation was carried out using an Anti-GFP antibody (HT801, TransGen Biotech, China) with the ChIP-IT® Express kit (Active Motif; CA, USA; 53008) according to the manufacturer's protocol. Gene-specific primers used for ChIP–qPCR are provided in Supplementary Table S1.

### RNA-seq and data analysis

One-month-old *PagHB7a-OE*, *PagHB7a-KO*, and WT plants were first cultured in 1/4 Hoagland’s solution for 18 d and subsequently treated with 100 mM NaCl for 0 and 6 h, and then roots were collected for total RNA extraction. mRNA sequencing was performed using the Illumina X Ten platform (Anoroad, Beijing, China). Three biological replicates were included per treatment, with each replicate consisting of three pooled plants. Paired-end sequencing (150 bp read length) was conducted. Clean reads were aligned to the *Populus trichocarpa* v3.0 reference genome using HISAT2 with default parameters^[55]^. Read counts per gene were obtained using HTSeq-count^[56]^. Differential gene expression analysis was performed with the edgeR package, considering genes with a *p-*value < 0.05 as significantly differentially expressed^[57]^. Gene Ontology (GO) enrichment analysis was carried out using the GOstats package in Bioconductor, with an adjusted *p-*value < 0.01 as the significance threshold^[58]^.

### Accession number

Genes from this article can be found with the accession numbers: *PagHB7a* (Potri.014G103000), *PagHB7b* (Potri.002G176300), *PagAREB1-2* (Potri.002G125400), *PagAREB1-3* (Potri.009G101200), *PagAREB1-4* (Potri.014G028200), *PagABF3* (Potri.004G140600), *PagRD26* (Potri.001G404100), *PagNAC072* (Potri.011G123300), *PagSTZ* (Potri.001G295500), *PagSTZ1-h4* (Potri.009G089400), *PagLEA* (Potri.002G165000), *PagPUB19* (Potri.015G074200), *PagPUB79* (Potri.012G078900), *PagRBOH* (Potri.003G159800), *PagNCED3* (Potri.001G393800), *PagNCED5* (Potri.011G112400), *PagCYP707A1* (Potri.004G235400).

## Results

### *PagHB7a* is significantly induced by salt stress and ABA

Based on transcriptome data from salt-stress experiments, two genes were identified, designated *PagHB7a* and *PagHB7b*, which were rapidly and significantly induced in poplar stems under salt treatment (Supplementary Fig. S1a)^[59]^, suggesting their potential role in salt stress response. *PagHB7a* and *PagHB7b* are homologs in *Populus* with 98% sequence similarity and belong to the HD-ZIP I subfamily. Similar to the Arabidopsis *ATHB7* gene, both genes contain a highly conserved DNA-binding homeodomain (HD), and an adjacent leucine zipper domain (LZ) (Supplementary Fig. S1b). Since *PagHB7a* exhibited more pronounced induction under salt stress, this gene was focused on to further investigate its function in salt stress tolerance.

**Figure 1:**
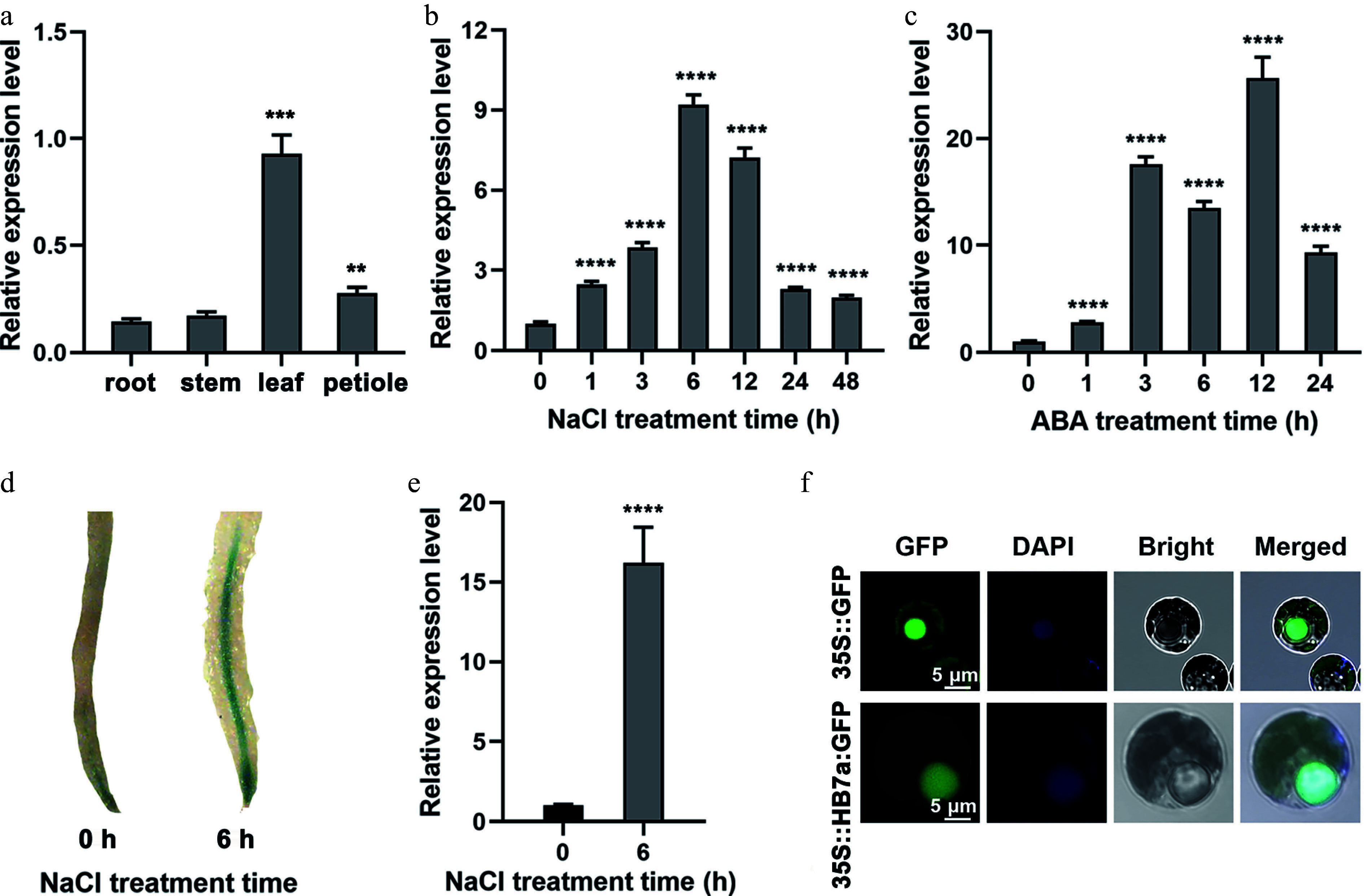
Expression profiles of *PagHB7a* in *Populus*. (a) Relative expression levels of *PagHB7a* in different tissues analyzed by RT-qPCR. Relative expression of *PagHB7a* in total roots of 84K poplar under (b) 100 mM NaCl, and (c) 200 μM ABA (c) treatments for the indicated times. (d) Histochemical GUS staining of roots from *ProPagHB7a::GUS* transgenic plants under 100 mM NaCl treatment. (e) RT-qPCR analysis of *GUS* expression level in total roots of *ProPagHB7a::GUS* transgenic plants under 100 mM NaCl treatment. (f) Subcellular localization of PagHB7a-GFP in 84K poplar protoplasts; nuclei were stained with DAPI (4′,6-diamidino-2-phenylindole). In (a)–(c) and (e), error bars represent ± SD (*n* = 3). Asterisks indicate significant differences compared to the 0 h control (Student's *t*-test): * *p* < 0.05, ** *p* < 0.01, *** *p* < 0.001, **** *p* < 0.0001. Error bars represent SD values (*n* = 3).

To further characterize the expression pattern of *PagHB7a*, RT-qPCR was performed to examine the expression of *PagHB7a* in various tissues, and under different treatments. Results showed that *PagHB7a* was expressed in all tissues examined, with relatively lower expression in roots (Fig. 1a). Under NaCl treatment, *PagHB7a* expression was significantly induced in roots, increasing nearly 10-fold within 6 h (Fig. 1b). Given the key role of abscisic acid (ABA) in plant stress responses, the response of *PagHB7a* to ABA was also examined. The results showed that ABA treatment strongly induced *PagHB7a* expression in roots, with a 26-fold increase observed after 12 h (Fig. 1c). *β*-Glucuronidase (*GUS*) histochemical staining of *ProPagHB7a::GUS* transgenic poplar plants further confirmed that the expression of *PagHB7a* was significantly induced in roots after NaCl treatment (Fig. 1d). Consistent with this, RT-qPCR analysis revealed that the expression of the GUS reporter gene increased more than 15-fold after 6 h of NaCl treatment (Fig. 1e). In agreement with its predicted function as a transcription factor, the PagHB7a-GFP fusion protein was localized exclusively in the nucleus (Fig. 1f).

### PagAREB1s directly activate the expression of *PagHB7a*

**Figure 2:**
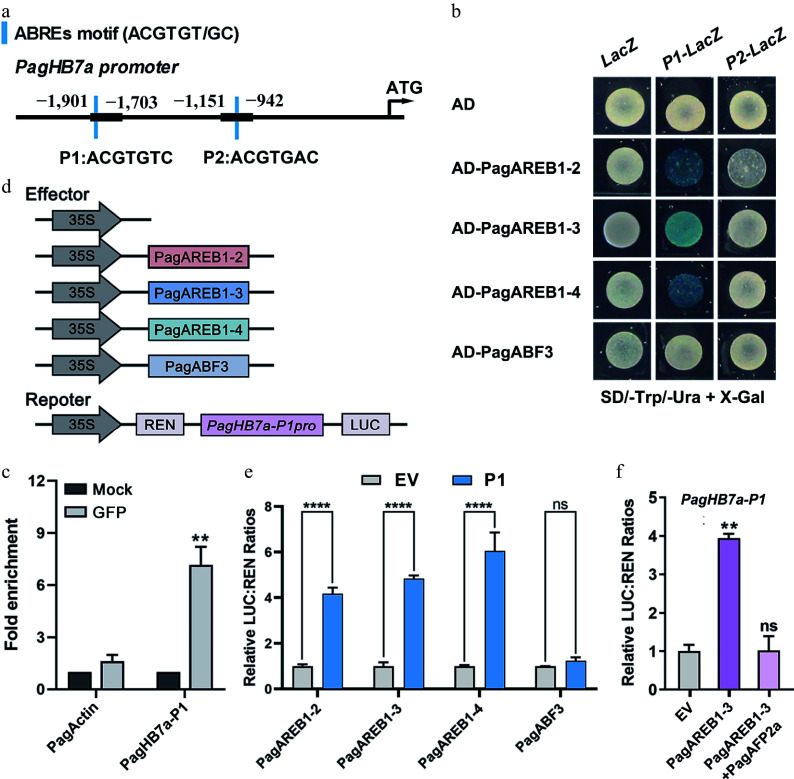
*PagHB7a* is directly bound and activated by PagAREB1s. (a) Schematic diagram of the *PagHB7a* promoter. Blue bars indicate ABRE cis-elements; P1 and P2 denote promoter fragments containing ABRE motifs. (b) Yeast one-hybrid (Y1H) assay shows that PagAREB1-2, PagAREB1-3 and PagAREB1-4 bind to the *PagHB7a* promoter region containing P1. (c) ChIP-qPCR demonstrates that PagAREB1-3 binds to the *PagHB7a* promoter region containing P1 *in vivo*. Statistical comparisons between the Mock (no anti-GFP), and anti-GFP groups were performed using Student's *t*-test. ** *p* < 0.01. (d) Schematic representation of effector and reporter constructs used in dual-luciferase assays. (e) Dual-luciferase assay shows that PagAREB1-2, PagAREB1-3, and PagAREB1-4 activate the expression of firefly luciferase (*LUC*) gene driven by the *PagHB7a* promoter region containing P1 in tobacco leaves. (f) Dual-luciferase assays show that PagAREB1-3 activates *LUC* expression driven by the *PagHB7a* promoter region containing P1, while co-expression of PagAFP2a inhibits the activation. The relative LUC:REN ratio in control samples (transformed with the empty vector, EV) was normalized to a value of 1. Error bars represent SD (n = 3). Asterisks indicate significant differences (Student's *t*-test): ** *p* < 0.01; **** *p* < 0.0001.

The *AREB*/*ABF* gene family plays a critical role in ABA-mediated responses to salt and drought stresses^[60]^. Analysis of the *PagHB7a* promoter identified two ABRE motifs (P1 and P2) within the 2,000 bp region upstream of the start codon (Fig. 2a). We previously identified four AREB genes (*PagAREB1-2*, *PagAREB1-3*, *PagAREB1-4*, and *PagABF3*) that were significantly upregulated under salt stress and co-expressed with *PagHB7a* within the same co-expression module^[59]^. To determine whether *PagHB7a* is a direct target of these transcription factors, yeast one-hybrid (Y1H) assays were performed. The results showed that PagAREB1-2/PagAREB1-3/PagAREB1-4 bound directly to the *PagHB7a* promoter region P1, while PagABF3 exhibited no binding (Fig. 2b). Chromatin immunoprecipitation (ChIP)-qPCR assays further confirmed the binding of PagAREB1-3 to the P1 region of the *PagHB7a* promoter (Fig. 2c). Subsequently, transient expression experiments were performed to test whether PagAREB1s could activate the expression of *PagHB7a* transcription (Fig. 2d). The results revealed that the relative luciferase activity driven by the *PagHB7a-P1* promoter was significantly enhanced when co-transformed with the PagAREB1-2, PagAREB1-3, and PagAREB1-4 effector. In contrast, the relative luciferase activity was not significantly different from the empty vector (EV) when co-transformation with PagABF3 (Fig. 2e). Our previous studies found that PagAFP2a, a protein that negatively regulates salt stress, antagonizes the function of PagAREB1-3 by repressing the transcriptional activity of PagAREB1-3 at its target genes in salt stress signaling^[27]^. Therefore, whether PagAFP2a negatively affects the transcriptional activation of *PagHB7a* was tested by PagAREB1-3 with dual-luciferase transient expression assays. The results showed that the co-transformation of PagAFP2a with PagAREB1-3 significantly inhibited the activation of *PagHB7a* by PagAREB1-3 (Fig. 2f). Collectively, these results demonstrate tha**t** PagAREB1-2, PagAREB1-3, and PagAREB1-4 directly bind to the *PagHB7a* promoter and activate its expression.

### PagHB7a is a positive regulator of salt tolerance and ABA sensitivity in *Populus*

**Figure 3:**
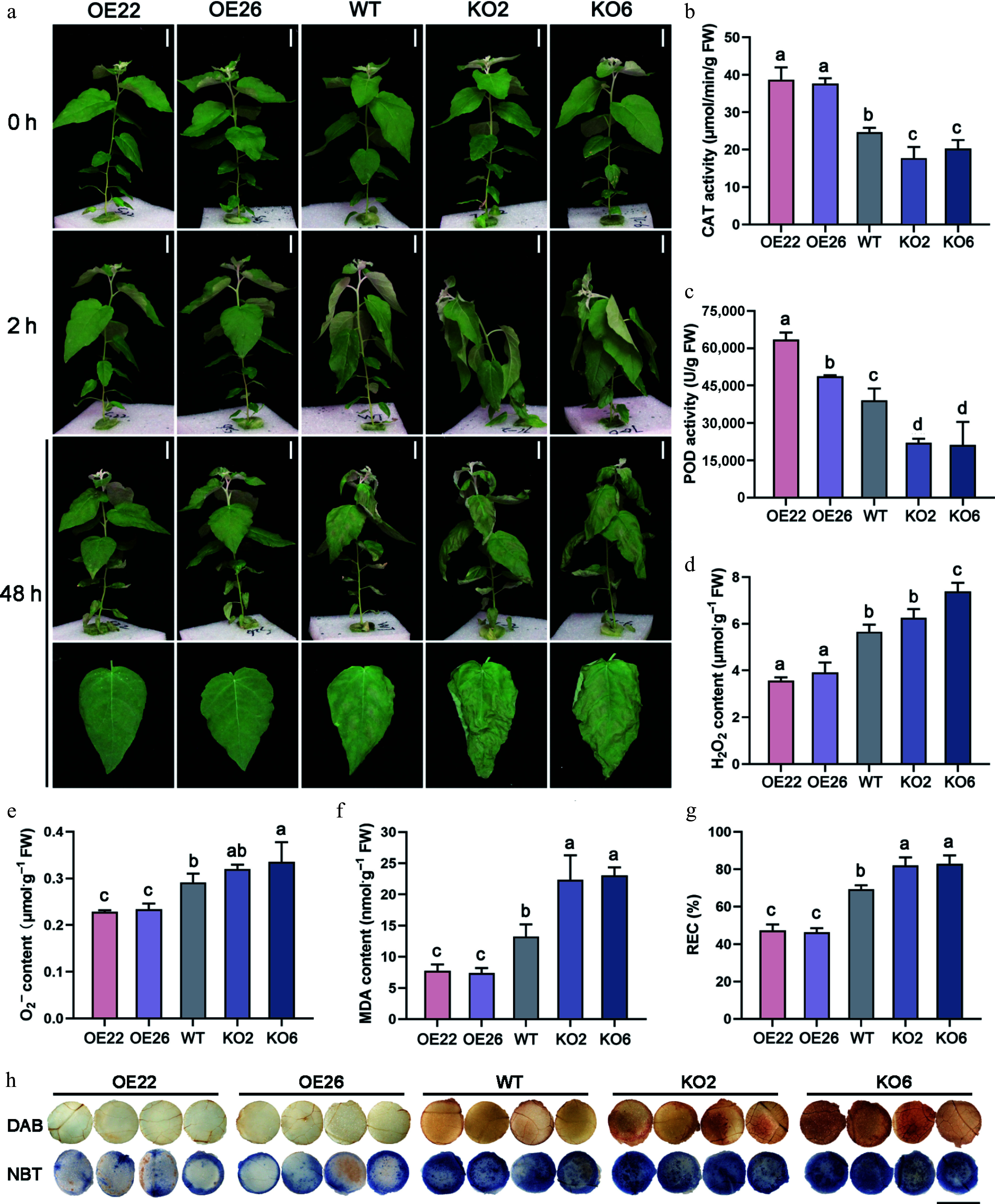
PagHB7a is a positive regulator of short-term salt tolerance in poplar. (a) Phenotype of *PagHB7a-OE*, WT, and *PagHB7a-KO* plants before and after treated with 150 mM NaCl. Scale bars, 5 cm. The (b) catalase activity (CAT), (c) peroxidase activity (POD), (d) H_2_O_2_ content, (e) O_2_·^−^ content, (f) malondialdehyde (MDA) content, and (g) relative electrical conductivity (REC) in *PagHB7a-OE*, *PagHB7a-KO*, and WT plants with 150 mM NaCl treatment. Error bars represent ± vSD (*n* = 3). Statistical significance was determined by one-way ANOVA with Tukey's post hoc test. Different letters represent significant differences. (h) Histochemical staining of reactive oxygen species using DAB (3,3'-diaminobenzidine), and NBT (nitroblue tetrazolium) in leaves of plants under 150 mM NaCl treatment. Scale bars = 6 mm.

To investigate the function of PagHB7a in response to salt stress, *PagHB7a* overexpression lines (*PagHB7a-OE*), and CRISPR/Cas9-mediated knockout mutants (*PagHB7a-KO*) in poplar were generated. Two *PagHB7a-OE* (OE22, OE26) lines with high transgene expression level, and two homozygous *PagHB7a-KO* (KO2, KO6) lines were selected for further analysis (Supplementary Fig. S2). First, the short-term salt stress responses of *PagHB7a-OE*, WT, and *PagHB7a-KO* plants were tested in liquid-culture with 150 mM NaCl. After 2 h of salt treatment, *PagHB7a-KO* plants exhibited stem tip bending and leaf curling, whereas WT plants showed only moderate leaf drooping. *PagHB7a-OE* plants were minimally affected, displaying only slight drooping. This phenotypic divergence became more pronounced after 48 h, with severe wilting and necrosis appearing in most *PagHB7a-KO* leaves, and WT plants showed noticeable damage. In contrast, *PagHB7a-OE* plants remained largely undamaged (Fig. 3a).

Salt stress induces excessive accumulation of reactive oxygen species (ROS), causing oxidative damage to conductive cells and consequently leading to elevated malondialdehyde (MDA) content, and increased relative electrical conductivity (REC)^[61]^. Thus, MDA content, REC values, and the ROS-scavenging activities of catalase (CAT) and peroxidase activity (POD) serve as key indicators of plant salt tolerance. Quantification analysis found that under salt stress, *PagHB7a-OE* plants exhibited significantly higher CAT and POD activities, along with markedly lower levels of H_2_O_2_, O_2_·^–^, MDA, and REC compared to WT plants (Fig. 3b–g). In contrast, *PagHB7a-KO* plants showed reduced CAT and POD activities, and elevated accumulation of ROS (H_2_O_2_ and O_2_·^–^), MDA content, and REC (Fig. 3b–g). Additionally, ROS staining analysis was performed on leaf tissues from each plant genotype using 3,3'-diaminobenzidine (DAB) and nitroblue tetrazolium (NBT). Consistently, the leaves of *PagHB7a-KO* displayed stronger ROS staining while the leaves of *PagHB7a-OE* displayed weaker staining compared to the WT under salt stress (Fig. 3h). These results suggested that PagHB7a positively regulates salt tolerance in poplar.

**Figure 4:**
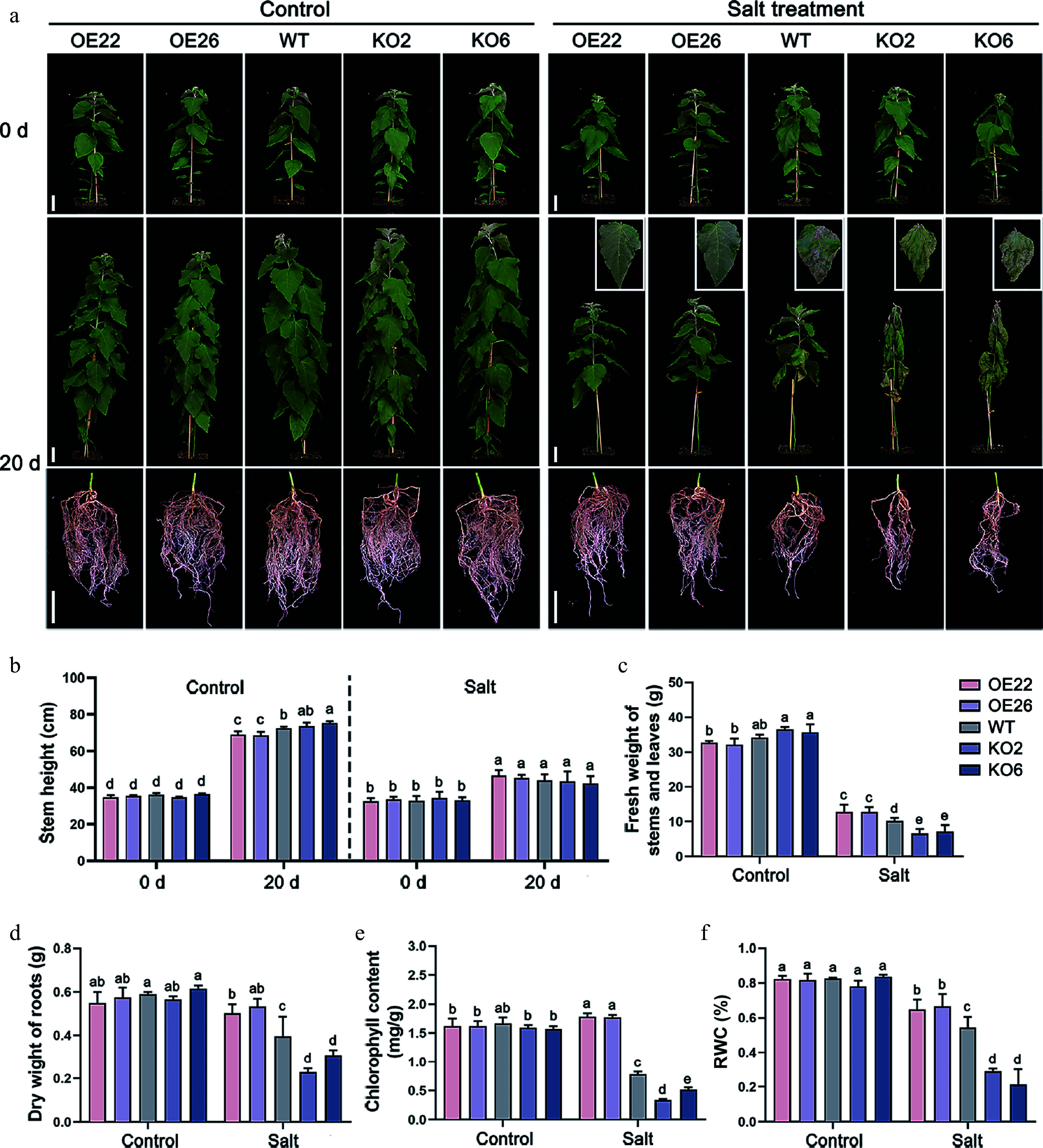
PagHB7a is a positive regulator of long-term salt tolerance in poplar. (a) Phenotypes of *PagHB7a-OE*, WT, and *PagHB7a-KO* plants treated with or without (Control) 100 mM NaCl treatment in soil. Scale bar = 5 cm. (b) Stem height of *PagHB7a-OE*, WT, and *PagHB7a-KO* plants under control conditions, or after treatment with 100 mM NaCl treatment. Quantification analysis of (c) fresh weight of stems and leaves, (d) dry weight of roots, (e) chlorophyll content, and the (f) relative water content (RWC) of *PagHB7a-OE*, WT, and *PagHB7a-KO* plants under control conditions or after treatment with 100 mM NaCl for 20 d. Statistical significance was determined by one-way ANOVA with Tukey's post hoc test. Different letters represent significant differences. Error bars represent SD values (*n* = 3).

To investigate the role of PagHB7a in response to long-term salt stress, we treated soil-grown *PagHB7a-OE*, WT, and *PagHB7a-KO* plants with 100 mM NaCl solution. After 20 d of salt treatment, *PagHB7a-KO* plants exhibited severe wilting and leaf desiccation, while WT plants exhibited moderate wilting. In contrast, the upper and middle leaves of *PagHB7a-OE* plants exhibited only slight drooping (Fig. 4a). Under control conditions (without NaCl), after 20 d of growth, *PagHB7a-OE* plants exhibited slight growth inhibition, with plant height reduced by 4.8% and 5.1% compared to WT, whereas *PagHB7a-KO* plants showed a slight increase in height (Fig. 4b). Prolonged salt stress eliminated these growth differences, resulting in similar plant heights across all lines. At the end of the treatment, several physiological indicators associated with salt stress were measured. These results showed that the *PagHB7a-OE* plants accumulated greater biomass (fresh weight of stems and leaves, and dry weight of roots) compared to WT under salt stress, while the *PagHB7a-KO* plants exhibited a reduction in biomass (Fig. 4c, d). Additionally, the chlorophyll content and the relative water content (RWC) in the *PagHB7a-OE* plants leaves were significantly higher than those in WT leaves under salt stress, whereas *PagHB7a-KO* plants showed the opposite trend (Fig. 4e, f). Under normal growth conditions (Control), no significant differences were observed in physiological parameters among all tested poplar lines (Fig. 4b–f). Together, these results further support that *PagHB7a* is a positive regulator of salt tolerance in poplar.

Given the central role of ABA signaling in abiotic stress responses and the significant induction of *PagHB7a* by ABA, its function in ABA sensitivity was investigated. Growth assays on medium containing 5 μM ABA revealed that *PagHB7a-OE* plants were hypersensitive, exhibiting significant reductions in root fresh weight and length, compared to WT (Supplementary Fig. S3a–S3c). Although the *PagHB7a-KO* plants showed a consistent trend toward reduced sensitivity, the difference was not statistically significant. Collectively, these results suggested that PagHB7a is a positive regulator of ABA signaling.

To assess the impact of PagHB7a on ABA homeostasis, the expression of ABA metabolic genes in the leaves of *PagHB7a* transgenic and WT plants following ABA treatment were further analyzed. In *PagHB7a-OE* plants, the expression of ABA biosynthesis genes *PagNCED3* and *PagNCED5*^[62]^ were significantly upregulated compared to WT, while the expression of *PagCYP707A1*^[63]^, which encodes a key enzyme in ABA oxidative catabolism, remained unaltered (Supplementary Fig. S3d). Conversely, *PagHB7a-KO* plants exhibited significant upregulation of *PagCYP707A1*, with no discernible change in *PagNCED3* or *PagNCED5* expression (Supplementary Fig. S3d). These results suggest that PagHB7a fine-tunes ABA homeostasis through coordinated regulation of both synthetic and degradative pathways.

### Transcriptome analysis of *PagHB7a* overexpression and knockout plants

**Figure 5:**
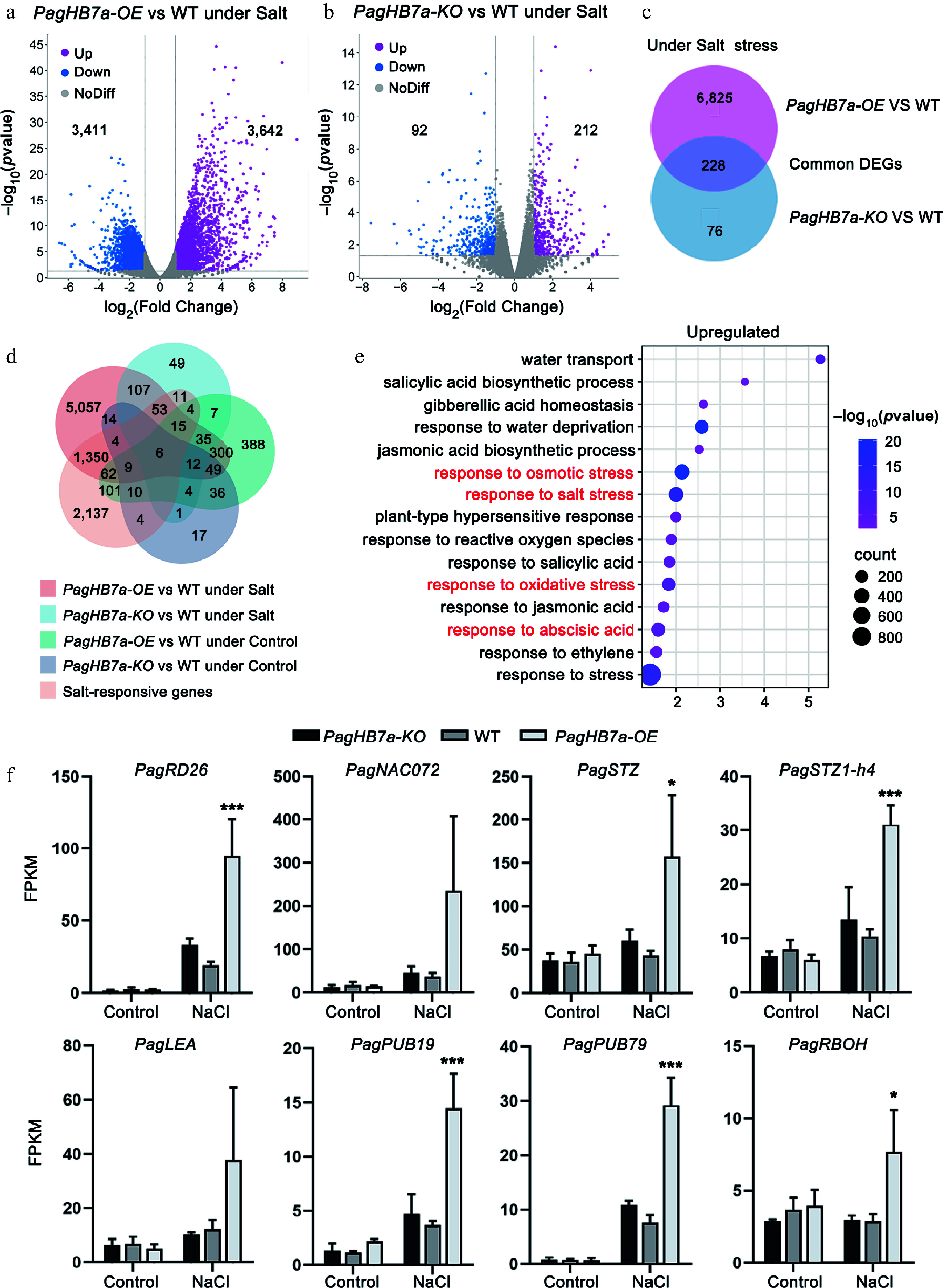
Transcriptome analysis of *PagHB7a-OE*, *PagHB7a-KO*, and WT plants. Volcano plots showing differentially expressed genes (DEGs) in (a) *PagHB7a-OE*, and (b) *PagHB7a-KO* plants compared to WT under NaCl treatment (*p*-adjust < 0.05, log_2_(Fold Change) ≥ 2.0). The *x*-axis represents log_2_ transformed gene expression level, and the *y*-axis represents the −log_10_ transformed *p*-adjust. Each dot represents a single gene. Purple and blue dots denote significantly upregulated and downregulated genes, respectively. Grey dots represent genes without significant changes in expression. (c) Venn diagram illustrating the overlap of DEGs between *PagHB7a-OE* and *PagHB7a-KO* plants under NaCl treatment conditions. (d) Venn diagram showing DEGs shared among five comparison groups. (e) Gene Ontology (GO) enrichment analysis of upregulated DEGs in *PagHB7a-OE* under NaCl treatment. Terms with large gene hit and low *p*-adjust. The size of the circles indicates the number of hit genes, and the color represents the significance level based on the adjusted *p*-value. (f) Expression of representative stress-responsive DEGs in *PagHB7a-OE*, *PagHB7a-KO*, and WT plants under 100 mM NaCl treatment for 6 h. Gene expression levels are shown as mean FPKM from RNA-seq data. Asterisks denote significant differences compared to values of WT, * *p* < 0.05; ** *p* < 0.01; *** *p* < 0.001.

To explore the regulatory network of PagHB7a in response to salt stress, RNA-seq was performed to profile transcriptomic changes in the roots of *PagHB7a-OE* (OE22), WT and *PagHB7a-KO* (KO2) plants. A total of 7,035 differentially expressed genes (DEGs) were identified between *PagHB7a-OE* and WT plants under salt stress (*p*-value < 0.05), comprising 3,642 upregulated and 3,411 downregulated genes (Fig. 5a, Supplementary Table S2). In contrast, only 304 DEGs were detected between *PagHB7a-KO* and WT plants under the same condition (*p*-value < 0.05), including 212 upregulated and 92 downregulated genes (Fig. 5b, Supplementary Table S3). Comparative analysis identified 228 common differentially expressed genes (DEGs) in *PagHB7a-OE* and *PagHB7a-KO* plants under salt stress conditions (Fig. 5c, Supplementary Table S4). A comparative analysis was further conducted using DEG sets from both *PagHB7a-OE* and *PagHB7a-KO* plants under control and salt-stressed conditions, alongside a reference set of 3,747 salt-responsive DEGs previously identified in the poplar salt-stress transcriptome (Supplementary Table S5)^[59]^. Integration across these five datasets revealed that 1,480 DEGs from salt-stressed *PagHB7a-OE* plants, and 89 DEGs from salt-stressed *PagHB7a-KO* plants overlapped with DEGs in salt-stressed WT plants (Fig. 5d, Supplementary Table S6, S7). Additionally, 488 DEGs were common between control and salt-stressed *PagHB7a-OE* plants, while 23 DEGs were shared between the two conditions in *PagHB7a-KO* plants. (Fig. 5d, Supplementary Tables S8, S9).

Gene Ontology (GO) analysis revealed that upregulated DEGs in *PagHB7a-OE* under salt stress were significantly enriched in pathways related to response to salt stress (GO: 0009651), response to abscisic acid (GO:0009737), response to osmotic stress (GO:0006970), and oxidative stress (GO:0006979) (Fig. 5e, Supplementary Table S10). Consistent with the high salt tolerance of *PagHB7a-OE* plants, key salt- and osmotic-stress-responsive genes (including *PagRD26*, *PagSTZ*, and *PagSTZ1-h4*)^[64,65]^ were significantly upregulated under salt stress. *PagNAC072*^[66]^ and *PagLEA*^[67]^, which were key genes involved in osmoregulation and ion homeostasis, also exhibited a consistent upregulation trend in *PagHB7a-OE* plants, further supporting the coordinated activation of the stress response network, even though these changes did not reach statistical significance (Fig. 5f). In contrast, *PagHB7a-KO* plants showed only marginal, non-significant changes in the expression of these genes after NaCl treatment (Fig. 5f). In addition, two genes involved in the ABA signaling pathway (*PagPUB19* and *PagPUB79*)^[68,69]^ were also upregulated in *PagHB7a-OE* plants, but only induced slightly in *PagHB7a-KO* plants after NaCl treatment (Fig. 5f). Furthermore, the expression levels of an ortholog of the respiratory burst oxidase homolog (*RBOH*) gene, a key player in systemic ROS signaling under salinity stress was analyzed. This gene was significantly upregulated in *PagHB7a-OE* plants, but remained unchanged in *PagHB7a-KO* plants^[70,71]^ (Fig. 5f). Together, the transcriptional data support the conclusion that PagHB7a positively regulates salt- and ABA-associated genes.

## Discussion

Understanding how plants respond to salt stress and balance growth with salt tolerance has long been a major focus of plant research^[30,72−76]^. HD-ZIP transcription factors are known to play crucial roles in conferring abiotic stress tolerance, with several *HB7* genes implicated in the regulation of plant responses to salt and drought stress^[28,41,43,77]^. In this study, it was demonstrated that *PagHB7a* is a positive regulator of salt tolerance in poplar. Overexpression of *PagHB7a* enhanced salt tolerance, while CRISPR/Cas9-generated mutants exhibited increased sensitivity to salt stress (Figs. 3a, 4a).

Abscisic acid (ABA) plays a central role in plant stress perception, particularly in salt-sensitive *Populus* species^[78]^. Building on previous identification of salt-responsive regulatory genes and co-expression modules in poplar^[59]^, it was found that *PagHB7a* expression is significantly induced under both salt and ABA treatments (Fig. 1b–e, Supplementary Fig. S1a), suggesting its potential involvement in ABA-mediated salt stress adaptation. AREB-type transcription factors, members of the bZIP family, typically function in abiotic stress responses through ABA-dependent pathways^[79−81]^, by binding to ABRE cis-elements associated with stress-responsive gene expression^[82−84]^. In this study, it was demonstrated through yeast one-hybrid (Y1H), ChIP–qPCR, and dual-luciferase reporter assays, that PagAREB1s binds directly to an ABRE-rich segment (P1) in the *PagHB7a* promoter and activates its expression (Fig. 2a–e). Furthermore, extending earlier reports that PagAREB1s-PagAFP2a forms a negative feedback loop to fine-tune salt-responsive gene activation^[27]^, it is shown that PagAFP2a also suppresses PagAREB1-3-driven transcription of *PagHB7a* (Fig. 2f).

Reactive oxygen species (ROS) levels, malondialdehyde (MDA) content, relative electrical conductivity (REC), and antioxidant enzyme activities are established biochemical indicators of plant salt tolerance^[45,85−87]^. Accordingly, these parameters were measured in *PagHB7a-OE*, *PagHB7a-KO*, and WT plants under short-term salt stress. Under salt stress, *PagHB7a-OE* plants exhibited significantly increased catalase (CAT) and peroxidase activity (POD), along with markedly reduced levels of ROS (H_2_O_2_ and O_2_·^−^), MDA, and REC, whereas *PagHB7a-KO* plants showed opposite trends (Fig. 3c–h). These findings suggest that PagHB7a enhances the enzymatic antioxidant capacity under salinity, thereby promoting redox homeostasis and membrane stability. This aligns with previous studies showing that modulation of *PagHB7a* expression influences ROS-scavenging ability and abiotic stress tolerance^[28,43,88]^.

Under normal soil conditions, *PagHB7a-OE* plants exhibited reduced growth compared to WT after 20 d, whereas *PagHB7a-KO* plants showed a slight increase in height (Fig. 4b). Notably, long-term salt stress alleviated the growth inhibition observed in *PagHB7a-OE* plants and the growth enhancement in *PagHB7a-KO* plants under control conditions (Fig. 4b). These findings suggest that PagHB7a fine-tunes growth responses under salinity, thereby modulating the trade-off between growth and tolerance. Moreover, *PagHB7a*-*OE* plants accumulated more biomass in both shoots and roots under salt stress than WT, whereas *PagHB7a-KO* plants exhibited suppressed growth and increased salt sensitivity, resulting in reduced biomass (Fig. 4b–d). These results strongly suggest that salt-induced PagHB7a plays a critical role in salt tolerance. It should be noted that due to the relatively short duration of the long-term salt treatment applied in this study, the phenotypic differences, although statistically significant, were relatively modest. Further field-based studies under long-term natural saline conditions will be essential to fully elucidate the role of PagHB7a in balancing growth and stress adaptation in poplar.

Through RNA sequencing, genes that were differentially expressed (DEGs) in *PagHB7a-OE* and *PagHB7a-KO* plants compared to WT plants under both salt stress and normal growth conditions were identified (Fig. 5a–c). Notably, the significantly lower number of DEGs in *PagHB7a-KO* plants, compared to *PagHB7a-OE* plants (304 vs 7,035) under salt stress, suggest functional redundancy within the HD-ZIP I transcription factor family. Given the high sequence similarity (98%), and co-induction under salt stress between *PagHB7a* and its close homolog *PagHB7b* (Supplementary Fig. S1), it is speculated that *PagHB7b* may compensate for the loss of *PagHB7a* in knockout plants, maintaining expression of critical downstream targets, and thus buffering the transcriptional impact of *PagHB7a* knockout. Such genetic redundancy is common in plant transcription factor families^[89−92]^ and helps ensure robustness in biological systems. Therefore, although *PagHB7a-KO* plants exhibit clear salt sensitivity, the full regulatory potential of *PagHB7a* is more evident from overexpression studies. Gene Ontology (GO) analysis revealed that under salt stress, the upregulated DEGs in *PagHB7a-OE* plants were significantly enriched in biological processes related to salt stress (GO: 0009651), response to abscisic acid (GO:0009737), response to osmotic stress (GO:0006970), and oxidative stress (GO:0006979) (Fig. 5e, Supplementary Table S10), which is consistent with the observed salt-tolerant phenotype and physiological changes. Furthermore, many genes involved in salt, osmotic, and oxidative stress responses, as well as ABA signaling, were significantly up-regulated in *PagHB7a-OE* plants under salt stress (Fig. 5f), confirming that PagHB7a acts as a positive regulator in the salt stress response.

**Figure 6:**
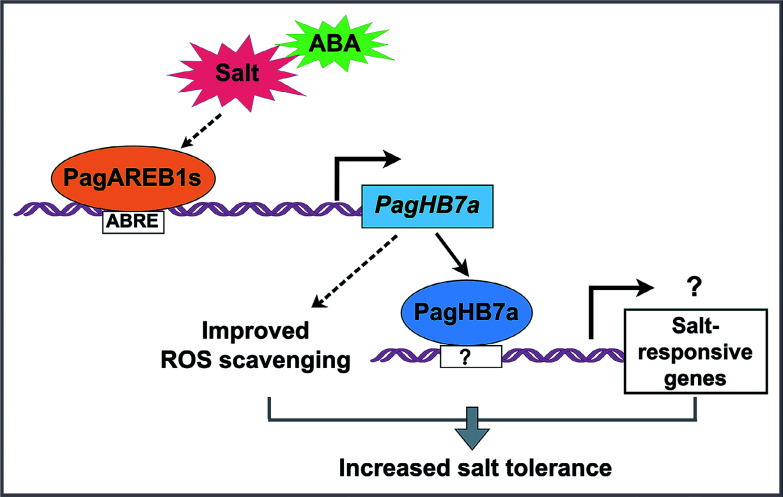
Proposed model for the regulatory role of PagHB7a in salt stress response. Under salt stress, PagAREB1s, key transcription factors within the ABA signaling pathway, directly bind to the promoter of *PagHB7a* and activate its expression. The upregulation of *PagHB7a* enhances reactive ROS scavenging capacity, thereby improving salt tolerance in poplar. Additionally, PagHB7a may also contribute to salinity adaptation through direct activation of unidentified salt-responsive genes.

A mechanistic model is proposed, illustrating the PagAREB1s-PagHB7a-mediated regulatory network underlying salt tolerance in poplar (Fig. 6). Under salt stress, *PagHB7a* expression is directly activated by PagAREB1s. The increased PagHB7a protein enhances salt tolerance by boosting ROS scavenging capacity. Furthermore, PagHB7a may also directly regulate certain salt-responsive genes by binding to unidentified cis-elements, thereby promoting transcriptional reprogramming and salinity adaptation. It would be of interest to further investigate whether PagHB7a interacts with other key regulators to form protein complexes that coordinately modulate salt tolerance in poplar.

## Conclusions

In this study, the HD-ZIP transcription factor PagHB7a was identified as a key positive regulator of salt tolerance in poplar. Its expression is directly activated by PagAREB1s, establishing it as a key downstream component of the ABA signaling pathway. Genetic analyses confirmed that *PagHB7a* overexpression significantly enhances tolerance to both NaCl and ABA treatment, whereas its knockout increased sensitivity. Furthermore, transcriptome profiling revealed that PagHB7a coordinates a molecular network governing responses to salt stress, ABA signaling, and oxidative stress. Future research should focus on identifying the direct transcriptional targets of PagHB7a and conducting their functional characterization. Overall, this research uncovers a key PagAREB1s-PagHB7a regulatory network, elucidates the molecular mechanism of PagHB7a-mediated salt tolerance, and provides a valuable candidate gene for breeding salt-tolerant poplar varieties.

## SUPPLEMENTARY DATA

Supplementary data to this article can be found online.

## Data Availability

The RNA-seq data underlying this article are available in the Genome Sequence Archive (GSA) at https://ngdc.cncb.ac.cn, and can be accessed with CRA032891.
