# Supplementary material for: The Class I HD-ZIP transcription factor PagHB7a functions as a positive regulator of salt tolerance in Populus
Source: For Res (Fayettev). 2025 Dec 31;5:e030. doi: 10.48130/forres-0025-0030 (PMC12982921; doi:10.48130/forres-0025-0030)
Supplement: Supplementary file 1 — Supplementary data to this article can be found online. [file forres-0025-0030-Supplementary.zip › 10.48130_forres-0025-0030-Suppl-FigureS1.pdf]

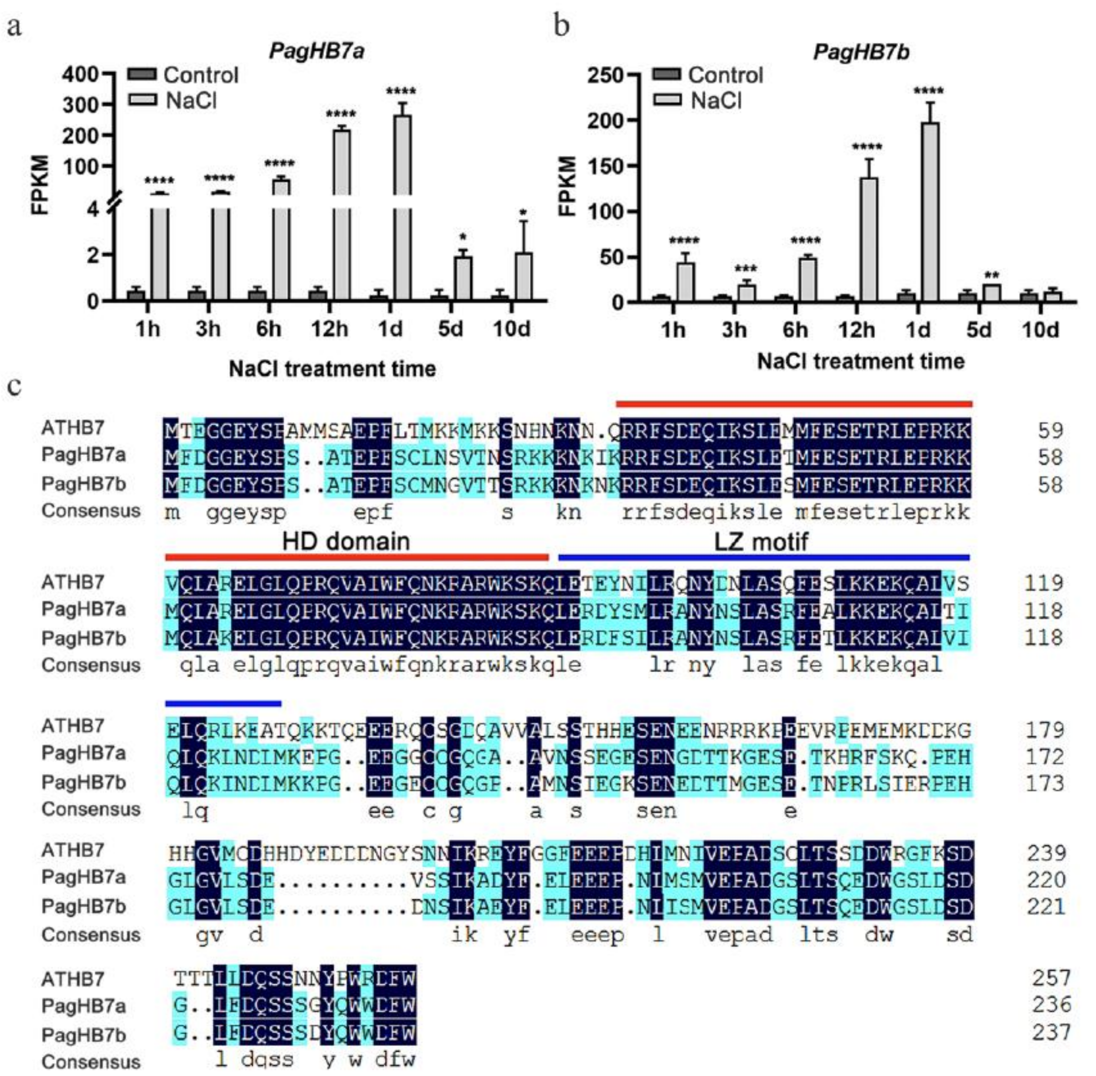

Figure S1. Characterization of PagHB7a in *Populus*. (a, b) Transcript levels of *PagHB7a* (a) and *PagHB7b* (b) in wild-type (WT) plants under NaCl treatment for the indicated durations. WT plants were treated with either Hoagland nutrient solution (Control) or 100 mM NaCl in Hoagland solution. Expression levels are presented as mean fragments per kilobase of exon per million fragments mapped (FPKM) from RNA-seq data. Error bars represent ±SEM of three biological replicates. Asterisks indicate significant differences compared to the Control. $*P < 0.05$; $**P < 0.01$; $***P < 0.001$; $****P < 0.0001$. (c) Amino acid sequence alignment of PagHB7s in *Populus* (*Populus alba* × *Populus glandulosa*) and AtHB7 in *Arabidopsis*. Identical and similar amino acids are shaded black and blue, respectively.
