# Supplementary material for: The Class I HD-ZIP transcription factor PagHB7a functions as a positive regulator of salt tolerance in Populus
Source: For Res (Fayettev). 2025 Dec 31;5:e030. doi: 10.48130/forres-0025-0030 (PMC12982921; doi:10.48130/forres-0025-0030)
Supplement: Supplementary file 1 — Supplementary data to this article can be found online. [file forres-0025-0030-Supplementary.zip › 10.48130_forres-0025-0030-Suppl-FigureS2.pdf]

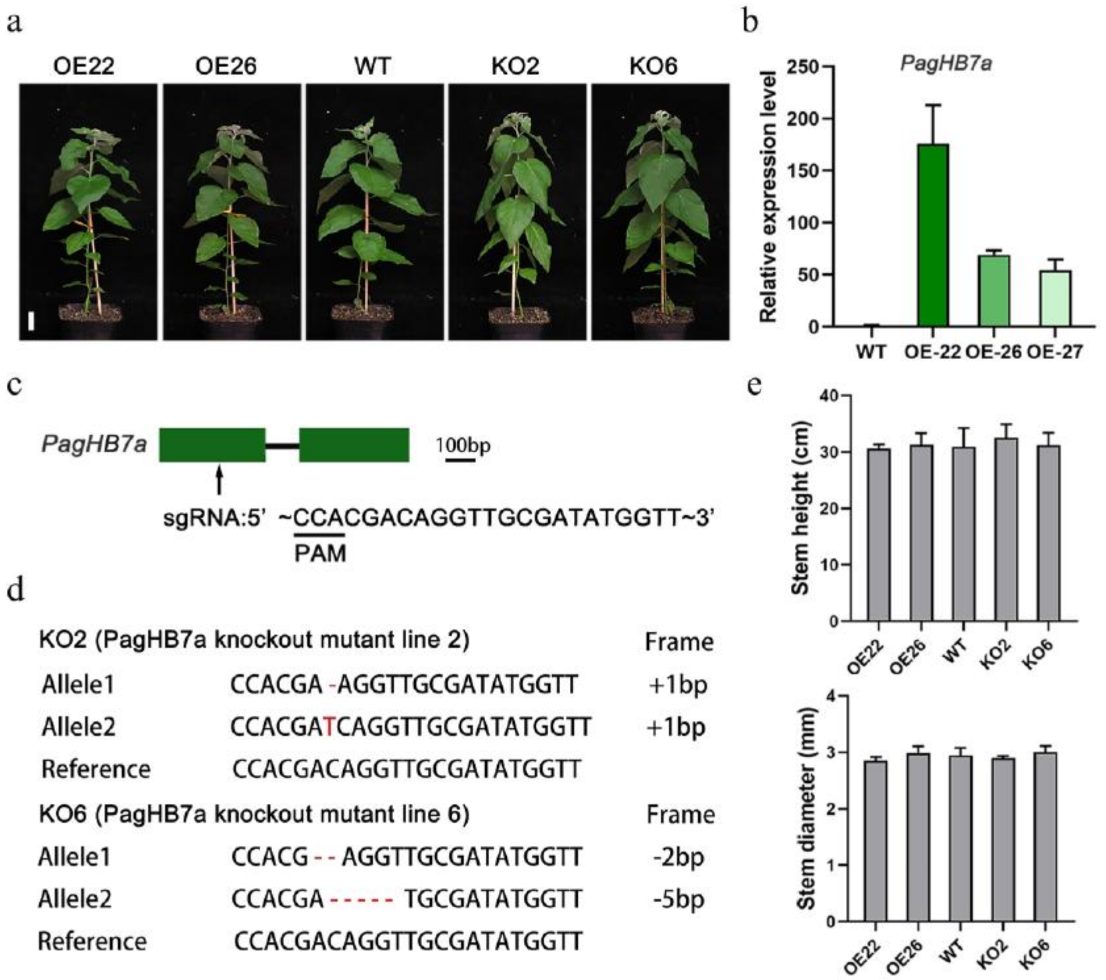

Figure S2. Identification of *PagHB7a* overexpression (*PagHB7a-OE*) and knockout (*PagHB7a-KO*) transgenic plants. (a) Phenotypic morphology of 2-month-old *PagHB7a-OE* and *PagHB7a-KO* plants. Scale bar, 3 cm. (b) RT-qPCR analysis of *PagHB7a* expression levels in overexpression transgenic and WT plants. (c) sgRNA location and nucleotide sequence in *PagHB7a* gene structure map. (d) Mutations in the sgRNA target of *PagHB7a* CRISPR/Cas9-mediated knockout mutant plants. Red characters indicate inserted or deleted nucleotides. The reference sgRNA target sequence is shown at the bottom. (e) Statistical analysis of stem height and stem diameter of *PagHB7a-OE*, *PagHB7a-KO* and WT plants. Error bars represent SD values (n = 3).
