# Supplementary material for: The Class I HD-ZIP transcription factor PagHB7a functions as a positive regulator of salt tolerance in Populus
Source: For Res (Fayettev). 2025 Dec 31;5:e030. doi: 10.48130/forres-0025-0030 (PMC12982921; doi:10.48130/forres-0025-0030)
Supplement: Supplementary file 1 — Supplementary data to this article can be found online. [file forres-0025-0030-Supplementary.zip › 10.48130_forres-0025-0030-Suppl-FigureS3.pdf]

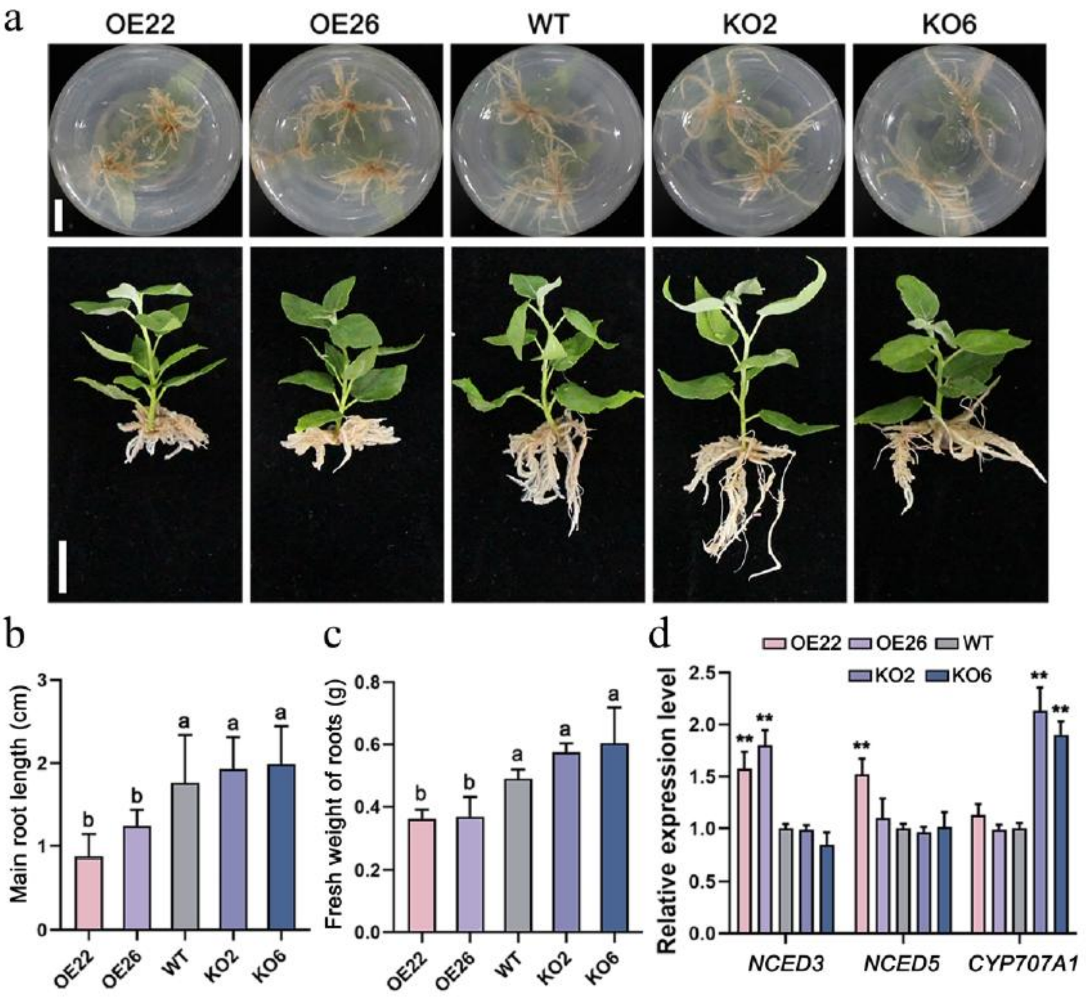

Figure S3. PagHB7a is a positive regulator in response to ABA treatment. (a) Phenotype of the 7-day-old *PagHB7a-OE*, WT and *PagHB7a-KO* plants treatment with 5 μM ABA treatment for 23 days, Scale bar, 1 cm. (b-c) Statistical analysis of the main root length (b), and fresh weight of roots (c) of *PagHB7a-OE*, WT and *PagHB7a-KO* plants under 5 μM ABA for 23 days. Error bars represent ±SD (n = 3). Statistical significance was determined by one-way ANOVA with Tukey's post hoc test. Different letters represent significant differences. (d) Relative expression level of *PagNCED3*, *PagNCED5* and *PagCYP707A1* were determined by RT-qPCR in the *PagHB7a-OE*, WT and *PagHB7a-KO* plants with 5 μM ABA treatment for 23 days, respectively. Asterisks indicate significant differences (Student's t-test): **$P < 0.01$.
